# An Acoustic Characterization of Prosodic Differences in Autism Spectrum Disorder and First-Degree Relatives

**DOI:** 10.1007/s10803-020-04392-9

**Published:** 2020-02-13

**Authors:** Shivani P. Patel, Kritika Nayar, Gary E. Martin, Kathryn Franich, Stephanie Crawford, Joshua J. Diehl, Molly Losh

**Affiliations:** 1 Roxelyn and Richard Pepper Department of Communication Sciences and Disorders, Northwestern University, 2240 Campus Drive, Frances Searle Building, #2-366, Evanston, IL 60208 USA; 2 Department of Communication Sciences and Disorders, St. John’s University, Staten Island, NY USA; 3 Present Address: Department of Linguistics and Cognitive Science, University of Delaware, Newark, DE USA; 4 LOGAN Community Resources, Inc, South Bend, IN USA; 5 Department of Linguistics, University of Chicago, Chicago, USA

**Keywords:** Autism spectrum disorder, Broad autism phenotype, Prosody, Acoustic

## Abstract

This study examined prosody through characterization of acoustic properties of the speech of individuals with ASD and their parents, during narration. A subset of utterances were low-pass filtered and rated for differences in intonation, speech rate, and rhythm. Listener ratings were minimally related to acoustic measures, underscoring the complexity of atypical prosody in ASD. Acoustic analyses revealed greater utterance-final fundamental frequency excursion size and slower speech rate in the ASD group. Slower speech rate was also evident in the ASD parent group, particularly parents with the broad autism phenotype. Overlapping prosodic differences in ASD and ASD Parent groups suggest that prosodic differences may constitute an important phenotype contributing to ASD features and index genetic liability to ASD among first-degree relatives.

## Introduction

Autism spectrum disorder (ASD) is a genetically-based neurodevelopmental disorder characterized by core deficits in social communication and restricted interests or repetitive behaviors (American Psychiatric Association 2013). Within the domain of social communication, prosody, which includes intonation modulation (changes in how ‘high’ or ‘low’ the voice sounds, based on rate of vocal fold vibration), rhythm (how evenly-timed syllables are in speech), and rate (how rapidly syllables are uttered in speech), has been noted as a key area of impairment in ASD (Peppé et al. 2006, 2007; Wells and Peppé 2003; Wells et al. 2004). It is important to note that prosody serves a variety of functions, all of which can impact communication. For example, stress and intonation can be used to encode grammatical information (e.g., the stress difference in the verb conTRAST versus the noun CONtrast), as well as pragmatic information (e.g., differentiating elements of discourse which are under discussion in the sentence “MARY saw the dog,” an appropriate answer to the question “Who saw the dog?” vs. “Mary saw the DOG,” an appropriate answer to the question “What did Mary see?”). Indeed, inappropriate use of stress in response to either question may disrupt communicative interactions. Furthermore, prosody conveys important information about speaker intent (e.g., sarcasm, persuasion, demand) and affect (e.g., joy, dislike) (Bachorowski 1999; Bachorowski and Owren 1995). Subtle abnormalities in intonation and rhythm have been found to adversely impact listeners’ ability to perceive and process speech in the general population (Bent et al. 2016).

In adolescence and adulthood, prosodic differences reported in individuals with ASD include atypical intonation and stress patterns, aberrant speech rate, lack of affective quality, and poor loudness control (Baltaxe and Simmons 1985; Baltaxe et al. 1984; Baron-Cohen and Staunton 1994; Fay 1969; Pronovost et al. 1966; Shriberg et al. 2001). While these atypical prosodic features don’t typically rise to the level of making speech unintelligible to a listener, they are among the first identifiable characteristics that create an impression of “oddness” among peers of individuals with ASD (Mesibov 1992; Van Bourgondien and Woods 1992). While prosodic deficits have been reported since the earliest delineations of ASD by Kanner and Asperger (Asperger and Frith 1991; Kanner 1943), McCann and Peppé’s (2003) review of sixteen studies of prosody in individuals with ASD revealed many inconclusive or contradictory findings across studies. For instance, whereas Fosnot and Jun (1999) reported atypical intonation patterns in individuals with ASD, another study (Baltaxe et al. 1984) found that intonation patterns did not differ from those of controls with typical development. Similarly, studies have reported both increased and decreased speech rate in individuals with ASD (Baron-Cohen and Staunton 1994; Shriberg et al. 2001). McCann and Peppé (2003) cite insufficient sample sizes, limited control data, and lack of standardized measures and sufficiently detailed methodology as explanations for the variability in findings. Furthermore, of those early studies reviewed, only two (Baltaxe et al. 1984; Fosnot and Jun 1999) made acoustic measurements of prosody, while others used subjective measures (i.e., perceptual judgments), which while clinically valid, offer only global characterization of differences.

More recent work applying acoustic analyses across a variety of communicative contexts has contributed to a more comprehensive understanding of specific prosodic atypicalities in individuals with ASD. For instance, examining speech of high-functioning individuals with ASD (HFA) and controls during a task involving retelling an emotional story, Edelson et al. (2007) found that the HFA group had significantly higher pitch overall and a wider pitch range compared to the control group. Diehl et al. (2009) also identified greater fundamental frequency (F0) variation during narrative production for both adolescent and child HFA groups compared to their respective control groups. Similarly, Nadig and Shaw (2012) found increased F0 range in speakers with HFA compared to an age- and language level-matched control group. In a study by Paul et al. (2008) involving the production of nonsense syllables, individuals with ASD demonstrated significantly less of a distinction in duration between stressed and unstressed syllables, as well as a pattern of increased F0 range for both stressed and unstressed syllables compared to controls, though this difference was not statistically significant. Using the Profiling Elements of Speech-Prosody in Communication (PEPS-C; Peppé and McCann 2003), Diehl and Paul (2013) found that individuals with ASD had a significantly greater F0 range than controls when using focus to highlight new information. However, the ASD group’s standard deviation (SD) and range of F0 did not differ from the control group in subtests involving expressing affect, producing appropriate utterance-final intonation for statements and questions, and signaling appropriate phrasal boundaries. In sum, although prior findings indicate variable differences in prosody across studies employing acoustic analysis versus perceptual judgments of prosody, together, prior work clearly demonstrates that prosody is impacted in ASD. It is likely that atypicalities along these acoustic and temporal dimensions contribute to impressionistic evaluations of speech in individuals with ASD as being overly ‘sing-songy’ (perhaps a reflection of greater F0 range in individuals with ASD) or machine-like (perhaps a reflection of lesser differentiation between stressed and unstressed syllables).

Differences in prosody have also been observed among clinically unaffected first degree relatives of individuals with ASD and may constitute a linguistic feature of the broad autism phenotype (BAP). The BAP refers to a constellation of subclinical language and personality features documented at elevated rates among relatives that parallel the defining features of ASD in quality (Losh et al. 2008; Losh et al. 2012a; Piven et al. 1997; Ruser et al. 2007). Such phenotypes are thought to reflect underlying genetic liability, and might afford better understanding of the range of phenotypic expression of ASD liability, and links to underlying biology. To this end, prior studies have shown a specific neuropsychological profile associated with the BAP in parents of individuals with ASD, where differences in social cognition and language processing abilities were evident in the subgroup of parents showing BAP features, whereas those without these subclinical phenotypes performed more similarly to controls (e.g., Losh et al. 2009; Nayar et al. 2018).

Subtle differences based on clinical-behavioral ratings of prosodic (also referred to as suprasegmental) language features (e.g., intonation and rhythm) have been reported in individuals with the BAP (Landa et al. 1992; Losh et al. 2008, 2012b; Piven et al. 1997). Further, a study exploring affective prosody perception in individuals with ASD and their siblings demonstrated that siblings exhibited some difficulty accurately perceiving emotion from prosody, though differences from controls were more subtle than those observed in the ASD group (Oerlemans et al. 2014). These findings suggest that prosodic differences could constitute an important feature of the BAP in unaffected relatives that indexes genetic liability to ASD. However, studies to date have only applied perceptual ratings to examine the acoustic properties of prosody in relatives of individuals with ASD, leaving unclear how these perceptually-based differences in prosody might bear out in objective acoustic measurements.

This study aimed to build on prior work by comparing acoustic profiles of prosody among individuals with ASD and their parents, with respective control groups. Overall, we tested the hypothesis that prosody is a linguistic marker of genetic liability to ASD by examining objective acoustic measures of prosody in individuals with ASD and their parents. Based on the literature reviewed above, we predicted that individuals with ASD and their parents would demonstrate overlapping areas of prosodic differences and that such differences would co-segregate with ASD severity in individuals with ASD and features of the BAP in parents. Additionally, we predicted that acoustic measurements from each group would relate to broader pragmatic (i.e., social) language atypicalities during conversational interactions in individuals with ASD and the BAP. Sex differences were also explored. Finally, listener-based perceptual ratings of prosody were collected in the ASD and ASD control groups to investigate how acoustic patterns might map to listeners’ perceptions of prosodic differences in individuals with ASD.

Based on prior literature characterizing prosody in individuals with ASD, we predicted that acoustic analyses would reveal differences in measurements of F0 variability (SD and range), rhythm, and rate in the ASD group. Given reports of prosodic differences in a subset of parents of individuals with ASD who exhibit features of the BAP, we predicted similar differences in acoustic measurements of F0 variability, rhythm, and rate in the ASD Parent group, and that these differences would be driven by parents with the BAP. Furthermore, we predicted that differences in acoustic measurements would be related to increased clinical-behavioral atypicalities, including increased pragmatic language violations in the ASD and ASD Parent groups, as well as ASD symptom severity in the ASD group. Finally, we predicted that the confluence of variables in which acoustic differences emerge would significantly contribute to listeners’ perceptual ratings of prosody in the ASD and ASD control groups.

## Method

### Participants

Study participants included 55 individuals with ASD (ASD group), 39 typically developing individuals with no family history of ASD (ASD control group), 96 parents of individuals with ASD (ASD parent group), and 48 parent controls without any family history of ASD (parent control group). Both the ASD and ASD control groups included children and adults. Parent groups included both parents when possible (ASD parent group: n = 28 couples; parent control group: n = 5 couples). Participants were recruited through a broader family-genetic study of ASD, which included individuals with ASD, their parents, and respective controls. Additional inclusionary criteria for all participants included having no history of brain injury, major psychiatric disorder, or known genetic syndrome or neurodevelopmental disorder (other than ASD), and being a native and fluent speaker of English. Furthermore, participants in either control group were excluded if they had first- or second-degree relatives with ASD, related genetic disorders (e.g., fragile X syndrome) or dyslexia. Individuals in the ASD parent group were excluded if they had a diagnosis of ASD.

Diagnosis of ASD was confirmed for all individuals in the ASD group with research reliable administration and scoring of the Autism Diagnostic Observation Schedule-2nd Edition (ADOS-2; Lord et al. 2000), as well as the Autism Diagnostic Interview-Revised (ADI-R; Lord et al. 1994) when time permitted. The ADOS-2 was also administered to individuals in the ASD control group to rule out ASD. ADOS-2 Overall, Social Affect, and Restricted and Repetitive Behaviors (RRB) calibrated severity scores were used to determine ASD severity (Hus et al. 2014; Lord et al. 2012).

The Wechsler Abbreviated Scale of Intelligence (WASI; Wechsler 1999), Wechsler Adult Intelligence Scale-Third or Fourth Editions (WAIS; Wechsler 1997, 2008), or the Wechsler Intelligence Scale for Children-Fourth Edition (WISC-IV; Wechsler 2003) were used to assess IQ for all participants. Table 1 summarizes the overall age, full-scale IQ, verbal IQ, and performance IQ for each group. The ASD group had a significantly lower full-scale IQ, VIQ, and PIQ than the ASD control group (*t*s > − 2.63, *ps* < .01). The ASD parent group had a significantly lower performance IQ and higher mean chronological age than parent controls (*t*s > − 2.18, *p*s < .03).

**Table 1:** Group characteristics

	ASD group	ASD control group	ASD parent group	Parent control group
	M (SD)	M (SD)	M (SD)	M (SD)
	Range	Range	Range	Range
Males:females	45:10^a^	19:20	38:58	20:28
Chronological age	16.57 (6.62)	18.99 (5.21)	46.67 (7.67)*	43.00 (9.87)
	6.45–35.10	12.38–32.03	28.38–65.76	25.95–63.89
Full-scale IQ	104.22 (12.03)*	115.45 (12.03)	111.09 (11.41)	114.81 (11.36)
	83.00–131.00	89.00–142.00	85.00–136.00	85.67–136.00
Verbal IQ	105.10 (13.79)*	117.50 (11.36)	109.41 (12.23)*	111.20 (12.85)
	82.00–146.00	93.00–142.00	80.00–132.00	82.00–138.00
Performance IQ	102.88 (14.58)*	111.08 (14.19)	110.40 (11.10)	115.08 (12.93)
	68.00–131.00	79.00–143.00	83.00–133.00	86.00–148.00

Significant differences (*p *< .05) between the ASD Group or ASD Parent Group and their respective control groups are indicated with *

*ASD* Autism Spectrum Disorder, *M* Mean, *SD* standard deviation

^a^Sex distribution in the ASD Group largely reflects sex bias noted in the prevalence of ASD (Werling and Geschwind 2013; Fombonne 2009; Idring et al. 2015)

### Narrative Elicitation

The 24-page wordless picture book, *Frog, Where Are You?* (Mayer 1969), was used to elicit spontaneous narratives from all participants across groups. This story has been used extensively in previous studies of narrative discourse in typically developing populations, as well as those with developmental disorders including ASD (Capps et al. 2000; Diehl et al. 2006; Losh and Capps 2003; Tager-Flusberg and Sullivan 1995). The story is about the adventures of a boy and his dog as they search for the boy’s missing pet frog. Participants were asked to narrate the story as each page was presented to them on a computer screen. All narrations were recorded using either a Blue Snowball USB microphone or a Logitech USB Desktop Microphone (980186-0403). The microphone was positioned approximately 8 inches from the participant’s mouth during the narration.

### Utterance Selection

In order to obtain relatively comparable speech samples from participants’ narratives, the first utterance from each page of the narrative was used in analysis, provided that the first utterance of a page did not meet exclusionary criteria consistent with criteria used in prior studies (i.e., the utterance contained character speech, a question, unfinished words, fewer than two words, an interruption by the examiner, or was unintelligible, directed towards someone else in the room and not related to the narrative, or abandoned; e.g., Shriberg et al. 2001). Importantly, the use of these exclusionary criteria promoted consistency in utterance length and type (e.g., no questions) across participants. Complete utterances with greater than two words were necessary for analysis of utterance-final F0 excursion size (described below). Additionally, these criteria ensured that included utterances were part of the narration rather than included as part of an interrupting conversation or other interaction. In cases where exclusionary criteria were met, the next utterance from the same page was chosen. If another utterance from the same page was unavailable or all utterances within the page met exclusionary criteria, an utterance from another page within the same section of the book (i.e., beginning, middle, or end) was selected. This resulted in a maximum of 24 utterances from each narration, each with a structure that typically corresponded to a maximal intonational phrase (i.e., the highest unit of structure within linguistic models of the prosodic hierarchy; Selkirk 2009, 2011). A minimum of 20 qualifying utterances were included from each participant. All audio recordings were analyzed using Praat (Boersma and Weenink 2017; http://www.fon.hum.uva.nl/praat/; version 6.0.29), a program for acoustic analysis of speech signals.

### Automatic Alignment of Speech Samples

The onset and offset of selected utterances, which were assessed by finding the points in the acoustic signal where a participant began and finished an utterance, were manually marked using TextGrids in Praat and subsequently automatically aligned at the level of words and phones (i.e., speech sounds) using FAVE (Forced Alignment and Vowel Extraction; Rosenfelder 2013).^1^ Each file was manually checked for inconsistencies and hand-corrected where necessary by the authors (all trained in phonetic analysis). Due to poor recording quality which prevented proper alignment, one adolescent subject and one parent subject were excluded from analysis, which is reflected in the sample sizes reported above. A Praat script with pitch tracking ranges dependent on speaker age and sex (see Table 2) was implemented in order to minimize pitch tracking errors in Praat. The script extracted F0 (in Hz) within each force-aligned utterance with a timestep of 0.01 s. Logarithmic transformation was applied to F0 values to approximate the scale on which pitch is perceived. After applying the logarithmic transformation, the mean, SD, and range of F0 for each utterance was calculated. Range of F0 was calculated by subtracting the maximum and minimum F0 values obtained from each utterance. Mean F0 is a measure of the rate of vocal fold (also known as “vocal cord”) vibration and is the physical correlate of pitch, or how “high” or “low” an individual’s voice is. The SD and range of F0 measure the extent to which an individual’s pitch varies during speech. Additionally, the F0 range (in Hz) of the final word of each utterance was calculated via Praat script as a measure of ‘excursion size’, or overall change in F0 (whether rising or falling) in utterance-final position (subsequently referred to as utterance-final F0 excursion size). Z-score transformation was applied to normalize data within the ASD, ASD control, ASD parent, and parent control groups.

**Table 2:** Fundamental frequency detection ranges

	Minimum	Maximum
Males 11 years and younger	130	400
Males 12–18 years of age	70	400
Males 19+ years	70	250
Females (all ages)	130	400

Speech rate for each utterance was automatically calculated using a script in *R* statistical software. Using input from the force aligned TextGrid files, the script computed the number of vocalic intervals (representative of the number of syllables) per utterance and divided these totals by the duration of each utterance, resulting in a measure of syllables per second (including within-utterance pauses).

Speech rhythm was calculated based on Normalized Pairwise Variability Index (nPVI; Grabe and Low 2002), a measure of durational variability between pairs of syllables in an utterance. The measure is calculated based on the following equation:

$$nPVI = 100 \times \left[ {\sum\nolimits_{k - 1}^{m - 1} {{{\left| {\frac{{d_{k} - d_{k + 1} }}{{d_{k} + d_{k + 1} /2}}} \right|} \mathord{\left/ {\vphantom {{\left| {\frac{{d_{k} - d_{k + 1} }}{{d_{k} + d_{k + 1} /2}}} \right|} {(m - 1)}}} \right. \kern-0pt} {(m - 1)}}} } \right]$$

The equation calculates the absolute value of the difference in duration *d* of adjacent syllables divided by the mean duration of the two syllables. These quotients are then averaged for the utterance^2^ and multiplied by 100. This method is used for evaluating relative changes in duration across syllables while controlling for effects of speech rate. Following the procedures of (Low and Grabe 1995, 2000), nPVI was calculated for individual utterances, as opposed to across all utterances. A lower nPVI indicates less variability in duration across syllables (i.e., a more uniform or staccato rhythm). We calculated nPVI for each utterance in the analysis, which typically corresponded with an intonational phrase.^3^ To ensure that any differences in nPVI across groups were not driven solely by differences in the use of utterance-initial fillers, which tend to be longer in duration than typical words and are also known to be utilized less by individuals with ASD (Gorman et al. 2016), utterance-initial fillers (i.e., “um” and “uh”) and utterance-initial syllables with a duration of greater than two standard deviations above the mean (300 ms) were deleted, as these elongated syllables can function in a similar way to fillers in delaying the onset of more regular, rhythmic speech (Clark and Fox Tree 2002). Henceforth, utterance-initial “um” and “uh” and elongated syllables will be collectively described as “fillers,” unless otherwise specified. Finally, utterances for which speech rate or nPVI were greater or less than two standard deviations from the mean were treated as outliers and removed. This resulted in a trimming of less than 5% of the total data.

### Clinical-Behavioral Measures and Correlates

#### Assessment of the BAP Personality Features

The Modified Personality Assessment Schedule (MPAS; Tyrer 1988) was used to assess the presence of personality traits of the BAP in the ASD parent group. Participants were asked a series of questions regarding personality traits that constitute the BAP, including social reticence, rigidity, and untactfulness. All interviews were rated by two coders, who were blind to participant group. Inter-rater reliability was 81%. Scores range from 0 (trait absent) to 2 (trait definitely present). Individuals were characterized as BAP(+) if they scored a 2 on the Social, Rigid, or Untactful traits of the MPAS. BAP(−) status was assigned when coders did not endorse traits on any of those subscales (scores < 2). These personality features are thought to mirror in quality the core domains of impairment in individuals with ASD, and have been shown to reliably distinguish ASD relatives from controls (Losh et al. 2008; Piven et al. 1997).

#### Pragmatic Language Ability

Pragmatic language skill was assessed using the Pragmatic Rating Scale-School Age (PRS-SA; Landa 2011) for individuals in the ASD and ASD control groups and using the Pragmatic Rating Scale (PRS; Landa et al. 1992) for individuals in the parent groups. The PRS-SA was rated from semi-structured interactions from the ADOS-2. Coders blind to group rated these interactions for pragmatic language features on a three-point scale as absent (0), unknown (1), or present (2) with an average item-level reliability of 76%. The PRS was coded based on a semi-structured conversational interview (Life History Interview) in which an examiner asks the participant a series of questions about their social relationships, family, academic achievements, and occupation. As in the PRS-SA, two independent coders blind to group status rated videos of these interactions on a three-point scale as absent (0), mildly present (1), or clearly present (2) for specific pragmatic language features, which include subjective measures of prosody such as intonation patterns and speech rate. Average item-level reliability for the PRS was 86%. Both the PRS-SA and PRS include several items that comprise sub-domains of pragmatic skill, including a suprasegmental domain that includes ratings of atypical intonation, speech rate, and volume. Additional domains contributing to overall pragmatic language skill assessed on the PRS-SA include: (1) presupposition/theory of mind, which involves understanding the perspective of the listener to decide what and how information should be shared; (2) discourse management, which includes topic initiation, maintenance, and conversational reciprocity; and (3) non-verbal communicative behaviors, such as gestures, affect, and proxemics. Additional domains on the PRS include management of listener expectations, such as providing clarification as needed and reciprocating during conversation, and dominating conversation, which includes overly detailed language use and topic preoccupation.

### Listener-Based Perceptual Ratings

Listener-based perceptual ratings were conducted on a subset of utterances produced during narrations from the ASD and ASD control groups. The third, eighth, and fourteenth utterance from each participant’s narration was selected to represent utterances from the beginning, middle, and end of the picture book. Audio recordings of each of the selected utterances were low-pass filtered from 0 to 400 Hz to preserve prosodic information from the signal while removing higher frequencies important for speech intelligibility. Each utterance was rated on an 11-point Likert scale from − 5 to 5 on variables of intonation (i.e., “flat” to “overly variable/sing-songy,” respectively), rate (i.e., “too slow” to “too fast,” respectively), and rhythm (i.e., “too staccato/choppy sound pattern” to “overly variable sound pattern,” respectively), with ratings of 0 indicating “typical.” To determine if ratings of the ASD group’s intonation, rate, and rhythm differed from ratings of the ASD control group, all ratings were transformed and analyzed as deviant from a rating of “typical.” After rating the intonation, rate, and rhythm, of each utterance, raters provided a judgement about how likely (definitely not, probably not, probably yes, definitely yes) it was that the speaker had ASD (“ASD likelihood”). For the purpose of planned analyses, ASD likelihood was recoded to a binary variable. Raters included 14 individuals ages 21–30 years with varying levels of experience with individuals with ASD (e.g., no direct experience, direct or indirect experience within an educational environment, interventionist, involvement with ASD research, family member with ASD). Raters with four or more years of experience working with individuals with ASD were classified as “expert raters” (n = 6) for subsequent analyses of perceptual ratings of prosody to determine if prior experience with individuals with ASD influenced ratings.

### Analysis Plan

A series of mixed effects linear regression models were conducted in the ASD and parent groups separately, investigating mean F0, SD of F0, range of F0, utterance-final F0 excursion size, speech rate, and nPVI were fitted using the *lme4* package (Bates et al. 2014) for *R* statistical software. All models including the ASD and ASD control groups included fixed effects of group, utterance length (number of syllables), and chronological age. Though the groups differed in IQ, the inclusion of full-scale IQ did not strengthen the model, so it was excluded. Models including the parent groups included fixed effects for group and utterance length (number of syllables). Age-related differences in F0 would not be expected in the parent groups, so age was not included in the model. The model for nPVI also included a fixed effect of speech rate and a two-way interaction between family diagnosis and speech rate, as speech rate and nPVI are known to covary. By-subject random intercepts were also included in all models, as well as random slopes corresponding to all fixed effects. To investigate potential sex-related differences in prosody, additional models separately comparing the males and the females in the child and parent groups were conducted using the same parameters described above. In addition to assessing overall group differences in the ASD parent and parent control groups, models were run to assess differences based on BAP status using Tukey multiple comparison tests. It is important to note that BAP status was not available for all participants due to missing data. The BAP(+) group included 44 individuals (males: 22; females: 22) and the BAP(−) group included 41 individuals (males: 12; females: 29). Finally, binary mixed effects logistic regression models were run to determine how well acoustic variables of mean F0, SD of F0, range of F0, speech rate, nPVI, and utterance-final F0 excursion size predicted diagnosis. By-subject random slopes corresponding to each of the three fixed effect variables were included in the model. For all models, continuous predictors were mean-centered to reduce collinearity. Kappa values for the models were all < 6, indicating that collinearity between predictors was low and unlikely to affect model results. All *p* values were estimated based on Kenward–Rogers degrees of freedom estimation using the package *lmerTest* for R. Finally, correlations between participant acoustic characteristics, clinical ratings of pragmatic language and ASD severity were examined.

Similar to analyses of acoustic measurements, a series of mixed effects linear regression models investigating listener-based perceptual ratings of intonation, rate, and rhythm were fitted using the *lme4* package (Bates et al. 2014) for *R* statistical software and included a fixed effect of group. Binary mixed effects logistic regressions were conducted to ascertain the effects of listener-based perceptual ratings of intonation, rate, and rhythm on raters’ estimates of actual group membership. For logistic regressions, values for predictor variables were centered around the ASD control group mean ratings. Analyses were conducted including responses from all raters and subsequently repeated including responses from expert raters only. By-rater and by-utterance-number random slopes corresponding to all three fixed effects were included in the model. Pearson correlations were conducted to assess associations between listener-based perceptual ratings of prosody and acoustic measurements. Statistically, in addition to *p* values < .05, *p* values < .10 were additionally reported in order to address the exploratory nature of some of our aims, and to increase transparency of reporting to inform further research with larger sample sizes. Importantly, findings with *p* values falling within the .05–.10 range are interpreted with caution.

## Results

### ASD and ASD Control Groups

#### Group Differences in Acoustic Measurements

**Fig. 1:**
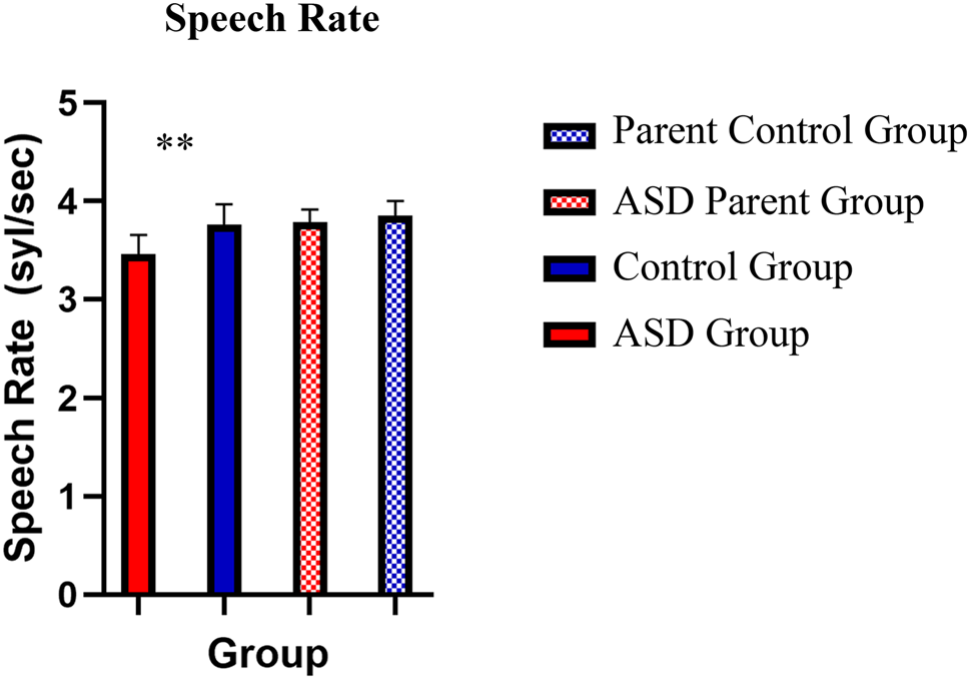
Speech rate across groups. Individuals with ASD exhibited significantly slower speech rate compared to controls. Error bars denote the standard error. ** denotes *p* < .01

No significant group differences were observed for mean F0 (*ß *=.22, *p *=.20), SD of F0 (*ß *=− .001, *p *=.96), or range of F0 (*ß *=− .06, *p *=.67). A marginally significant group difference was detected for utterance-final F0 excursion size, with the ASD group exhibiting a larger change in F0 in the utterance-final position (*ß *=− .12, *p *=.05). Additionally, a main effect of group for speech rate (*ß* − .59, *p *< .01) revealed a slower speech rate among individuals with ASD (see Fig. 1). There was no effect of group for nPVI (i.e., rhythm; *ß* = 1.74, *p* = .48), indicating no group difference in paired syllable durations. When examining acoustic prosodic differences in males only, males with ASD demonstrated a larger utterance-final F0 excursion size than controls (*ß *=− .20, *p *< .05), but no differences in mean F0 (*ß *=.11, *p *=.56), SD of F0 (*ß .*01, *p *=.95), range of F0 (*ß *=− .08, *p *=.70), or nPVI (*ß *=2.27, *p *=.48). Males with ASD demonstrated slower speech rate than controls (*ß *=− .56, *p *<.05). In analyses of females only, there was a marginally significant main effect of group on mean F0 (*ß *=.46, *p *=.09) such that females with ASD exhibited a lower mean F0 compared to female controls. Additionally, females with ASD demonstrated a marginally larger utterance-final F0 excursion size than female controls (*ß *=− .17, *p *=.07). There were no significant group differences in SD (*ß *=.24, *p *=.39), range of F0 (*ß *=.06, *p *=.85), speech rate (*ß *=− .25, *p *=.88), or nPVI (*ß *=2.69, *p *=.64). Mixed effects binary logistic regression indicated no combination of acoustic variables were significant predictors of ASD group membership.

#### Group Differences in Listener-Based Perceptual Ratings

**Fig. 2:**
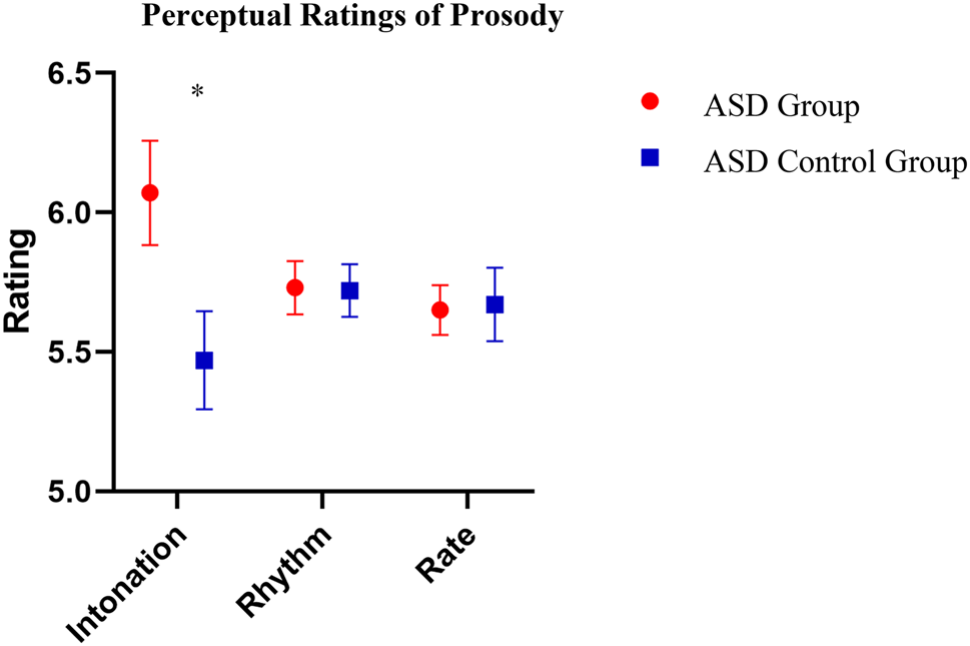
Perceptual ratings of prosody in ASD and ASD Control groups. Listeners identified significantly atypical intonation, but not rhythm or rate in the ASD group. Error bars denote the standard error. * denotes *p* < .05

A significant group difference was observed for ratings of intonation (*ß *= − .60, *p *=.03; see Fig. 2). For ratings by expert raters only, a main effect of group for intonation (*ß *= − .69, *p *=.03) was detected, along with a marginal effect of group for ASD likelihood (*ß *=− .09. *p *=.05) such that expert raters accurately identified individuals with a diagnosis of ASD as being more likely to have ASD based on the excerpted acoustic sample. Non-expert raters showed no such distinction (*ß *=− .04. *p *=.37). Raters (expert and non-expert) showed no differences in ratings of rate or rhythm (all raters, rate: *ß *= .04. *p *=.72, rhythm: *ß* = − .05. *p *=.72; expert raters only, rate: *ß *= − .01, *p *=.98, rhythm: *ß *= − .12, *p *=.44).

A binary logistic regression was conducted to determine the types of perceptual ratings based on the short excerpted acoustic samples that best predicted group membership. Findings indicated that raters’ perceptions of atypical speech rate and the likelihood of ASD diagnosis both significantly predicted ASD group membership (*β* = − .306, z = − 3.372, *p* < .001; *β* = .474, *z* = 2.935, *p* < .01, respectively). Ratings of intonation were not a significant predictor of ASD group membership (*β* = − 0.005, *z* = − .10, *p* = .92), nor were ratings of rhythm (*β* = .06, *z* = 1.11, *p* = .26). The model correctly predicted 91% of utterances from individuals in the ASD group and 14% of the control group’s samples, giving an overall percentage correct prediction rate of 59%. Thus, while the model showed quite high sensitivity, specificity was low.

#### Relationships Between Acoustic Measurements and Listener-Based Perceptual Ratings

Pearson correlations revealed significant positive associations between F0 variability (SD of F0 and utterance-final F0 excursion) and listener-based perceptual ratings of intonation (*r*s > .42, *p*s < .01), rhythm (*r*s > .33, *p*s < .05), and rate (*r*s > .28; *p*s < .05). Additionally, range of F0 was positively correlated with listener-based perceptual ratings of intonation (*r* = .32, *p *< .05). Finally, speech rate was positively associated with perceptual ratings of rate and rhythm (*r*s > .33; *p*s < .05).

### ASD Parent and Parent Control Groups

**Fig. 3:**
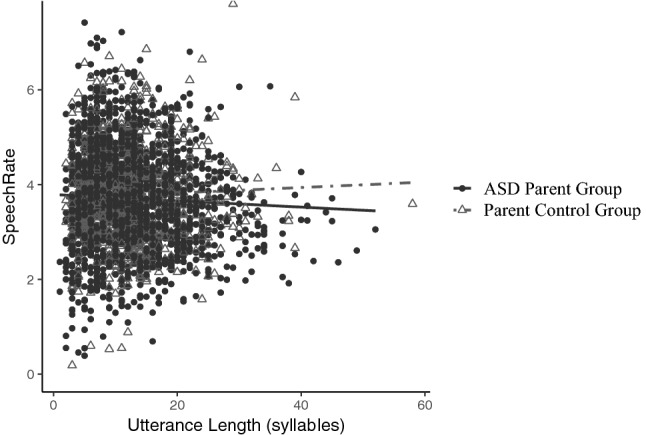
Interaction between speech rate and utterance length in the ASD parent and parent control groups. The ASD parent group showed significantly slower speech rate at higher utterance lengths compared to the parent control group (*p* < .05)

Significant parent group differences were observed for mean F0 (*ß *= − .46, *p *< .001) and range of F0 (*ß *= .28, *p *=* .*01), with the ASD parent group exhibiting a lower mean F0 and higher range of F0 compared to the parent control group. The ASD parent and parent control groups did not differ in SD of F0 (*ß *= .05, *p *=.68) or utterance-final F0 excursion size (*ß *= .07, *p *=.15). While there was no group difference in speech rate (*ß *= − .05, *p* = .44), there was an interaction between group and utterance length (*ß *= − .06, *p* < .05), such that parents of individuals with ASD showed greater slowing of speech rate as utterance length increased (see Fig. 3). There were no significant group differences on nPVI (*ß *=.08, *p* = .88).

When examining acoustic prosodic differences in fathers only, no significant group differences were detected for mean F0 (*ß *=− .31, *p *=.62), SD of F0 (*ß *=* .*0003, *p *=1.00), range of F0 (*ß *=− .02, *p *=.96), speech rate (*ß *=− .17, *p *=.42), or nPVI (*ß *=2.56, *p *=.34). However, fathers of individuals with ASD demonstrated a marginally smaller utterance-final F0 excursion size than control fathers (*ß *=.13, *p *=.08). Analyses of mothers revealed that mothers of individuals with ASD exhibited a significantly lower mean F0 and greater range of F0 compared to control mothers (*ß *=− .47, *p *<.001; *ß *=.31, *p *<.01, respectively). No significant group differences in SD of F0 (*ß *=.04, *p *=.79) or utterance-final F0 excursion size (*ß *=.05, *p *=.33), speech rate (*ß *=− .15, *p *=.41), or nPVI (*ß *=1.07, *p *=.65) were detected in mothers.

### BAP Group Differences

Differences in parent groups for mean F0 were driven by the BAP(+) group (*ß *= − .58, *p *< .01). In particular the BAP group differences in mean F0 were driven by mothers with the BAP (*ß *=− .58, *p *=.02; fathers: *ßs *<.34, *ps *>.10). There was a marginal effect of BAP status on range of F0 (*ß *= .28, *p *< .09) such that the BAP(+) group had a marginally higher range of F0 compared to parent controls. This marginal finding did not appear to be driven by sex (mothers: *ßs *<.29, *ps *>.13; fathers: *ßs *<.09, *ps *>.96). BAP status did not significantly influence SD of F0 or utterance-final F0 excursion size (*ß*s < .08, *p*s> .18). Furthermore, sex differences by BAP group were not identified for SD of F0 (mothers: *ßs *<.07, *p *>.91; fathers: *ßs *<.14, *p *>.92) or utterance-final F0 excursion size (mothers: ßs < 3.0, ps > .71; fathers: *ß*s < 10.5, *p*s > .30). Analysis of the effect of BAP status on speech rate revealed a significant interaction between speech rate and utterance length, demonstrating greater slowing of speech rate with increased utterance length in the BAP(+) group compared to parent controls (*ß* = .08, *p* < .05). Differences between the BAP(+) and BAP(−) groups did not reach significance (*ß *= .04, *p* = 0.31). The interaction did not reach significance for either sex (mothers: *ß *= − .09*, p *= .11; fathers: *ß *= − .02*, p *= .63), though the relationship was stronger for mothers than for fathers. There were no significant differences in nPVI by BAP status overall (*ß*s<.36, *p*s > .50) or by sex (mothers: *ß *= .52*, p *= .53; fathers: *ß *= .24*, p *= .75).

### Associations Between Acoustic Measurements of Prosody, ASD Severity, and Pragmatic Language

#### ASD and ASD Control Groups

In the ASD group, decreased speech rate was correlated with increased overall and social affect symptom severity based on the ADOS-2 (*r* = − .33, *p* = .04 and *r *= − .39, *p *=* .*01, respectively*).* Similarly, decreased speech rate was correlated with increased overall score on the ADOS-2 in the ASD control group (*r *= − .79, *p* = .03). Increased F0 range and decreased speech rate in the ASD group, were correlated with greater impairment in pragmatic language features related to theory of mind (e.g., not providing sufficient background information) based on the PRS-SA (r = .295, p = .049; r = .36, p = .02, respectively). Increased range of F0 in the ASD group was positively correlated with greater impairments in the suprasegmental domain of the PRS-SA (*r *= .32, *p *= .03). F0 excursion in the ASD group was positively correlated with ASD symptom severity in restricted and repetitive behaviors, driven primarily by males with ASD, (*r *=.48*, p *<.01), as well as increased pragmatic language impairment based on the PRS-SA (*r *=.41*, p *<.01).

#### ASD Parent and Parent Control Groups

Increased nPVI in the ASD parent group was associated with increased pragmatic violations overall, based on the PRS (*r* = .32, *p* = .02). This association was driven by the dominating conversation factor (e.g., overly detailed, tangential; *r* = .34, *p* = .01). Decreased range of F0 in the ASD parent group, particularly in mothers, was associated with increased violations in pragmatic language features associated with the managing listener expectations factor (e.g., fails to reciprocate, vague) during the PRS (*r* = − .34, *p* < .01). There were no significant associations between acoustic variables of prosody and clinical-behavioral measures in controls.

## Discussion

The present study examined acoustic profiles of prosody in individuals with ASD and their parents during a narrative task, and examined relationships with clinical-behavioral features in individuals with ASD and among parents. Analyses also compared acoustic measures with listener-based perceptual ratings of prosody for individuals with ASD. Findings from analysis of acoustic measures in the ASD group were perhaps most revealing in the relatively few group differences that were evident, particularly in light of more robust differences that emerged in perceptual ratings for the ASD group, which were in line with prior literature using listener-based ratings (Baltaxe and Simmons 1985; Fine et al. 1991; Fosnot and Jun 1999; Landa et al. 1992; Losh et al. 2008, 2012b; Piven et al. 1997). For instance, no differences were observed between the ASD group and controls in key acoustic features such as F0 variability or rhythm (measured using nPVI). Objective acoustic measures were also minimally related to listener-based judgments, underscoring the complexity of the prosodic differences associated with ASD. That is, the clear distinctions in prosody observed by listeners (and repeatedly documented in prior studies) do not appear to be reflected in any basic, or readily measured set of acoustic properties. Acoustic features that differed in the ASD group were most notable in cases where differences were also detected among clinically unaffected parents, and driven by the subgroup of parents who demonstrated features of the BAP. These findings together may point towards a core set of acoustic features influenced by genetic liability to ASD.

Specifically, our study found speech rate to be an important variable distinguishing prosodic profiles in individuals with ASD and the BAP, supporting prior reports from perceptual ratings of speech in individuals with ASD and the BAP (Landa et al. 1992; Losh et al. 2012a). Findings that speech rate was overall slower for individuals with ASD, and also slower with increased utterance lengths in BAP(+) parents, could implicate cognitive processing differences in individuals with ASD and the BAP. Importantly, prior research in typically developing populations has shown associations between increased cognitive load and reduced speech rate (Griffin and Williams 1987; Huttunen et al. 2011). Further research is necessary to understand the various cognitive and social-pragmatic factors that may influence prosodic features, such as speech rate, in individuals with ASD and how these may interact with one another.

In addition to reduced speech rate, individuals with ASD exhibited increased utterance-final F0 excursion. While this differs from overall differences in intonation variability (e.g., SD of F0, range of F0) previously identified in individuals with ASD (Diehl et al. 2009; Edelson et al. 2007; Nadig and Shaw 2012), use of more dynamic utterance-final F0 excursion may be related to prior descriptions of individuals with ASD inappropriately or ineffectively using questioning and statement intonation patterns (Paul et al. 2005; Peppé et al. 2007). Furthermore, despite a lack of overall acoustic differences in F0 variability, listener-based perceptual ratings revealed differences in ratings of intonation, suggesting that listeners are perceiving intonation differences in individuals with ASD that may not be due exclusively to differences in the F0 measurements examined here. One possibility is that additional acoustic measures of F0 need to be examined, such as the relative timing of pitch rises and falls, in addition to overall measures of pitch variability. Another possibility, consistent with prior work (Van Santen et al. 2010), is that listener perceptions of disordered prosody do not map directly to acoustic measures. It is well known, for example, that pitch and timing interact such that changes in timing can be perceived as changes in pitch, and vice versa (Lake et al. 2014; Yu 2010). Furthermore, it may be that differences in perceptual ratings reflect the confluence of speech properties impacting a listener’s ratings, rather than any individual component of the speech signal. This is supported by associations identified between increased F0 variability (i.e., SD and range of F0) and listener-based perceptual ratings of increased atypicalities in intonation, speech rate, and rhythm. Ratings of ASD likelihood based on the individual’s intonation, rate, and rhythm differed based on a rater’s prior experience with individuals with ASD even though ratings of the aforementioned prosodic components did not differ between expert and non-expert raters. This further suggests that there may be additional speech characteristics, not necessarily contained within the prosodic components highlighted in this study (e.g., vocal intensity/volume, timing of pitch rises or falls), influencing ratings of ASD likelihood and that raters with more experience with individuals with ASD may be more attuned to these features. This conclusion is also supported by recent work by Redford et al. (2018) in which naïve listeners presented with short speech segments from individuals with ASD and typically developing controls identified the speech of individuals with ASD as atypical based on speech patterns related to speech motor control and voice quality, rather than prosodic components in particular. More specifically, listeners stated that they based their judgements on what they perceived to be slurred speech and differences in speech rate, leading Redford et al. (2018) to posit that intelligibility was largely responsible for discriminating the speech of an individual with ASD. Similarly, in the present study, ratings of speech rate were the strongest predictors of ASD group membership, suggesting that even when presented with filtered speech in which speech intelligibility is unpreserved, speech rate remains important in distinguishing atypical speech patterns.

Furthermore, in addition to a more slowed speech rate with increased utterance length, results revealed a reduced mean and increased range of F0 in parents of individuals with ASD, specifically those with the BAP. Interestingly, the pattern of reduced mean and increased range of F0 was driven by females with the BAP. While evidence of clear sex-specific differences in the expression of the BAP is limited (Rubenstein and Chawla 2018; Sasson et al. 2014; Seidman et al. 2012), recent work has demonstrated stronger associations between mothers with the BAP and their children on measures of language fluency compared to fathers with the BAP (Nayar et al. 2018). Results from the present study add to such evidence, perhaps suggesting a stronger maternal effect in the language-related phenotypes reflecting genetic liability to ASD. More broadly, lower mean F0 among females in the general population has been shown to index aspects of social identity, such as intellectual status or “nerdiness.” Females may use such aspects of their speech to signal related virtues, such as intellectual involvement over social status, while at the same time signaling a rejection of mainstream ideals of femininity (Bucholtz 2012). In the context of this broader literature, female-specific speech patterns observed in the present study may similarly be socially-driven. Indeed, females with ASD have been shown to have a greater ability to communicate and socialize than males (Hiller et al. 2014; Sedgewick et al. 2016), and ASD has also been associated with academic talent among individuals without intellectual impairment (Baron-Cohen et al. 2007). It has also been cautiously proposed that there is a genetic link between ASD and certain types of academic talent (e.g., mathematical aptitude; Warrier and Baron-Cohen 2016), suggesting that such links may also be evident in individuals with the BAP.

Associations between acoustic measures of prosody and clinical-behavioral features provided further insight into the role of prosody within the overall presentation of ASD and the BAP. In the ASD and ASD control groups, decreased speech rate was associated with greater overall ASD severity, particularly in the social affect domain for individuals with ASD. Furthermore, correlations between acoustic measures during narrative and clinical-behavioral ratings of overall pragmatic language ability during semi-structured interactions suggest that cross-contextual atypicalities present differently in individuals with ASD and parents of individuals with ASD. In the ASD group, speech rate and range of F0 were related to broader pragmatic language atypicalities, including intonation and theory of mind-related pragmatic language skills. Relatedly, parents’ speech rhythm and range of F0 were related to overall atypicalities in pragmatic language, suggesting that these prosodic elements may co-occur with or contribute to pragmatic language difficulties. Of note, however, is that group differences revealed increased range of F0 in the ASD Parent group while decreased range of F0 was associated with increased pragmatic language atypicalities in this group. This discrepancy may be due, at least in part, to contextual differences in the collection of these measurements as prosody was assessed during narrative and pragmatic language atypicalities were measured during a conversational task. Taken together, findings suggest that prosody plays a key role in the larger social communication atypicalities that are observed in these groups, and may be contributing to what is qualitatively perceived as an “odd” or somewhat “socially awkward” interaction.

### Clinical Implications

In light of a lack of specificity between perceptual ratings of prosody and acoustic differences in individuals with ASD, it may be beneficial to focus on multiple features of prosody (e.g., intonation modulation, rate, rhythm) rather than restricting focus to any singular feature. Results also support the use of a multi-method approach that includes both acoustic measurements and listener-based perceptual ratings of prosody. Overall, findings highlight the importance of targeting prosody in speech-language interventions and that such intervention may benefit overall pragmatic language abilities. Yet, limited prosody-focused interventions for individuals with ASD exist (Diehl and Paul 2009; Paul 2001), underscoring the need for collaboration between clinicians and researchers in this area. Indeed, acoustic differences in speech rate and utterance-final F0 excursion identified in the present study may serve as important objective benchmarks for speech-language interventions.

### Limitations and Future Directions

The present study assessed prosody in individuals with ASD and their parents within a specific context. To better understand patterns of strengths and challenges, as well as genetically meaningful phenotypes associated with prosody, it will be important for future work to systematically assess prosody in more and less structured tasks in individuals with ASD and their family members given that varying social and cognitive demands across contexts may impact prosody. While the present study made several contributions to the literature in terms of assessesing sex differences in prosody in individuals with ASD, future work should seek to include a larger sample of females to expand on the present findings. Importantly, though the present study identified slower speech rate in the ASD group overall, this finding appeared to be driven by males with ASD, suggesting that speech rate may be an area of relative strength for females with ASD. Additional evidence is necessary to support this conclusion and would have broader implications for speech-language interventions tailored for females with ASD, who are known to exhibit a different clinical presentation from males (Head et al. 2014; Mandy et al. 2012; Sedgewick et al. 2016; Van Wijngaarden-Cremers et al. 2014). Furthermore, the present study focused solely on prosodic *production*. However, further insight into the prosodic profile in individuals with ASD may be gained through investigation of *receptive* prosody skills.

## Conclusion

In sum, this study revealed some key prosodic differences in individuals with ASD and the BAP, using a comprehensive suite of acoustic analysis in relation to clinical-behavioral characteristics, as well as perceptual ratings of prosody in the ASD group. In particular, prosodic differences in speech rate appear impacted in both individuals with ASD and clinically unaffected relatives with the BAP, in addition to differences in mean and range of F0 in individuals with the BAP, particularly among females with the BAP. Complementary analyses on acoustic measures and listener-based perceptual ratings demonstrate complexity of the prosodic differences in individuals with ASD such that listeners’ access to the combination of features available in the speech signal distinguish individuals with ASD even when objective measurements of discrete prosodic components may not.

## Abbreviations

ASD: Autism spectrum disorder
BAP: Broad autism phenotype
F0: Fundamental frequency
nPVI: Normalized pairwise variability index

## Footnotes

1 FAVE uses modified acoustic models for English from the Penn Phonetics Lab Forced Aligner (p2fa; Yuan and Liberman 2008) to predict where words and phones begin and end within the acoustic signal. More information about forced alignment with FAVE can be found at https://github.com/JoFrhwld/FAVE/wiki/Using-FAVE-align.

2 nPVI is calculated on a by-utterance basis since speech rate is known to vary from utterance to utterance even in the speech of a single individual (Miller et al. 1984). Calculating the value for each utterance therefore gives us a more fine-grained measure of rhythmic variability for each participant.

3 In theoretical models of prosody (Nespor and Vogel 1986; Pierrehumbert and Beckman 1988; Selkirk 1986), the intonational phrase is typically posited as the highest level within a hierarchy of prosodic constituents.
